# The hospital as the only place to live: Long-term hospital stays in a tertiary hospital for infectious diseases in Manaus, Brazilian Amazon

**DOI:** 10.1371/journal.pone.0357832

**Published:** 2026-09-08

**Authors:** Talyson Aparício Gomes, Joseir Saturnino Cristino, Rafaela Nunes Dávila, Bianca Leite Pereira, Milena Gomes Ferreira, Giulia Lória Aquino, Amanda Braga Cunha, Felipe Nery Braga, Érica da Silva Carvalho, Jacqueline Sachett, Felipe Gomes Murta, Vinicius Azevedo Machado, Wuelton Monteiro

**Affiliations:** 1 School of Health Sciences, Amazonas State University, Manaus, Brazil; 2 Department of Teaching and Research, Dr. Heitor Vieira Dourado Tropical Medicine Foundation, Manaus, Brazil; 3 Duke Global Health Institute, Duke University, Durham, North Carolina, United States of America; University of Brasilia Faculty of Medicine: Universidade de Brasilia Faculdade de Medicina, BRAZIL

## Abstract

Long-term hospital stays represent a growing challenge for health systems, especially in tertiary hospitals that receive patients with clinically complex pathologies. This phenomenon is often related to social factors, such as abandonment, absence of caregivers and fragility of support networks, and the difficulty of relocation to intermediary institutions. This study aimed to understand the experiences and perceptions of family caregivers and health professionals about prolonged hospital stays in a referral hospital for tropical and infectious diseases in the Brazilian Amazon. This is a qualitative, exploratory study that is based on interviews with ten health professionals and eight family members of patients hospitalized for more than or equal to 180 days. The data were analyzed via thematic analysis. The analyzed cases show an overlap of vulnerabilities, including comorbidities, HIV/AIDS infection and fragility in family ties. The stigma associated with HIV/AIDS and the families’ poor financial conditions hinder care after discharge, making the hospital the only possible space for these patients to stay. The analysis identified five major themes: (1) social hospitalization and institutional dependence; (2) dynamics of family and social ties; (3) routine care and impacts on the lives of caregivers; (4) clinical complexity and obstacles that impede hospital discharge; and (5) the need for public policies and infrastructure. It is concluded that, in the absence of alternatives outside the hospital and effective intersectoral policies, the hospital begins to play a role beyond that of clinical care, and functions as a place to stay and the only space for social bonds for individuals in situations of extreme social vulnerability.

## Introduction

The definition of patients with long-term hospital stays is related to the extending of the expected period of hospitalization in a hospital. However, there is still no consensus on a temporal criterion for its characterization, since, globally, its definition may vary due to the reasons for hospitalization, clinical outcomes and logistical and structural factors of the different health systems [1–4]. Due to the clinical complexity of the cases and the scarcity of viable alternatives for discharge, patients in a long-term situation end up hospitalized for months, or even years. These individuals present a clinical evolution that is marked by alternating between acute phases and chronic stabilization stages, which are associated with the reason for their admission, and which demands continuous care due to their high dependence on health care [5–9].

The prolonged stay of patients in medium and high-complexity hospitals is often seen as a challenge for bed management, since, even without the need for specialized care, many remain hospitalized due to the lack of safe alternatives for discharge [4.810]. In this scenario, it is common to use the pejorative term bed blockers, which trivializes the complexity of the issue by highlighting only the merely operational problem [10]. The long-term hospital stay, far from being only a problem exclusively of bed management, has its origin in structural gaps that go beyond the limits of the health system, referring to broader public policies, such as better distribution of income and access to basic rights, such as education, housing and social assistance, especially when associated with populations in socioeconomic vulnerability [11].

The reintegration of patients to social and family life after hospitalization for long periods faces multiple challenges, among them are the complexity of clinical cases, dependence on mechanical ventilation and advanced life support, in addition to the risks associated with transportation to their city of origin. Added to this is the insufficient infrastructure to ensure the necessary care outside the hospital environment and, in some cases, abandonment by the family, which results in the refusal to assume responsibility for care at home [12,13]. Prolonged hospitalization also has significant repercussions on the family nucleus, since the patient’s withdrawal from social life and his seclusion in the hospital environment impose changes in the routine of the closest relatives. This impact intensifies when there is a constant need for face-to-face monitoring, which implies not only physical and emotional overload, but also the neglect of other daily responsibilities [5,14–16].

Long-term hospital stays can result in the so-called social hospitalization. Andrew and Powell (15) state that the concept of social hospitalization has been used to characterize situations in which prolonged hospitalization is not only due to an acute clinical demand, but also to social conditions that prevent the patient from returning home. In these cases, hospital admission and stay are motivated by the collapse of support networks, the absence of caregivers, insecurity in the home environment or the inability of the family to deal with the patient’s needs, factors that are often invisible in the care process. Social hospitalization should be understood as a multifactorial phenomenon, which involves not only aspects of the patient’s clinical status, but also factors related to the family, caregivers, support institutions and the sociopolitical context of each health system. The patient is inserted in a layered social structure, which includes from poorly managed chronic diseases, the physical and emotional exhaustion of caregivers, the absence of effective public policies to situations of persistent social exclusion, elements that, when combined, hinder hospital discharge and sustain prolonged stay [15,16].

The long-term hospital stay represents a challenge for health systems, especially in hospitals where the clinical complexity of patients is associated with social and structural obstacles that impede hospital discharge. Despite the relevance of this phenomenon, there are still few studies that investigate the profile of these patients and the context in which they are inserted. Therefore, this study aimed to understand the experiences and perceptions of family caregivers and health professionals regarding long-term hospital stays in a referral hospital for tropical and infectious diseases in the Brazilian Amazon.

## Materials and methods

### Statement of ethics

This study was approved by the Ethics Committee in Research Involving Human Beings of the Universidade do Estado do Amazonas, under CAAE: 71193123.8.0000.5016, detailing all the procedures that were carried out during its realization according to resolutions 466/12 and 510/16 of the National Health Council of Brazil. Only adult participants (≥18 years old) were included in this study. All participants received a detailed explanation of the study objectives and procedures and provided written informed consent prior to the interviews. All names, details, and interview excerpts were anonymized to ensure confidentiality. This investigation followed the consolidated criteria for reporting qualitative research (COREQ) [17].

### Study site

This study was developed in the city of Manaus, in the state of Amazonas, at the Fundação de Medicina Tropical Doutor Heitor Vieira Dourado (FMT-HVD), a Brazilian public hospital linked to the Unified Health System (SUS). The FMT-HVD is considered a national and international referral unit in the treatment of tropical and infectious diseases, in addition to being structured to meet the medium- and high-complexity demands of patients from the capital and interior of the state.

The hospital has 164 beds for hospitalizations of medium and high complexity, in addition to having an intensive care unit (ICU) that receives patients from the state capital and those from the interior who are transferred for specialized treatment. The hospital has an emergency room that operates on a full-time basis, in addition to providing outpatient care focused on tropical and infectious diseases. in the northern region of Brazil, Amazonas stands out for being the largest state in the country, covering an area of more than 1.5 million square kilometers. This geographical vastness, added to the predominance of tropical forest and the low population density in many regions, presents significant challenges for the supply of health services, especially regarding the access of residents of remote areas to highly complex treatment units, as is the case of the FMT-HVD.

The referral of patients from the interior to the capital is often mediated by the system of state regulation, which assesses the clinical complexity and organizes the transportation of patients by water, land or air routes. However, the predominance of rivers as the main travel routes means that many journeys are prolonged and logistically complex, requiring the mobilization of local and regional resources to ensure the arrival of patients at the referral center in a timely manner. After treatment at FMT-HVD, the counter-referral system assists patients in returning to their place of origin with guidance for follow-up and rehabilitation in less complex units.

### Characterization of the patients

A search was performed in the FMT-HVD databases, using the local information system Idoctor, to identify possible patients eligible for the study. Medical records were accessed for research purposes between April and June 2024. All data were fully anonymized by the institution’s data management team before being made available to the authors, in accordance with the ethical guidelines of the institutional Ethics Committee (CEP) and the Brazilian National Research Ethics Commission (CONEP). At no point did the authors have access to identifiable information. Considering the literature [1–4] and the local characteristics, we defined a long-term patient in our case as one with a duration equal to or greater than 180 days (6 months) and covered in this investigation hospitalized patients between the years 2010 and 2023.

The general characteristics of the patients with prolonged hospitalizations were analyzed regarding their clinical and sociodemographic data, such as age, sex, nationality, diagnosis, clinical picture, social evaluation and hospital outcome. A total of 286 medical records were considered eligible, and an individual evaluation of each case was performed, resulting in the exclusion of 93 open files due to outpatient care, 57 in which it was not possible to accurately determine the length of hospitalization due to failures in information systems, 67 that were unavailable for consultation and 18 that did not present sociodemographic data, clinical characteristics or information about the length of hospitalization. The study included 51 patients who met the criteria and whose medical records allowed their clinical and social characterization, as well as the identification of potential family members/caregivers throughout hospitalization. These patient-level data constituted a descriptive component and were not treated as the primary unit of the qualitative analysis.

### Informants

The study was initially presented to the institution’s Social Assistance Department, whose professionals had previous knowledge of patients with long-term hospital stays and assisted in identifying potential participants. Health professionals were eligible if they had worked regularly at the institution for at least two years and had direct or indirect experience in the care or management of these patients. Eligible participants included physicians, nurses, psychologists, social workers, and health managers.

Family caregivers were eligible if they were aged 18 years or older, had accompanied a patient during prolonged hospitalization, and agreed to discuss their caregiving experience. They had family ties with patients included in the study and were identified through information recorded in medical records and social assessments, with support from the institution’s professionals.

Intentional sampling was used to select participants whose experiences were directly relevant to the study objective and to the primary unit of qualitative analysis, which comprised the experiences and perceptions of family caregivers and health professionals. The first contact with potential participants was made by telephone, followed by a face-to-face explanation of the study and invitation to participate.

### Study design

This was a qualitative exploratory study conducted in a referral hospital for tropical and infectious diseases in the Brazilian Amazon. The primary unit of qualitative analysis consisted of the experiences and perceptions expressed by family caregivers and health professionals during the interviews. Clinical and sociodemographic data from patients’ medical records were used descriptively to characterize long-term hospitalization and contextualize the qualitative findings.

### Data collection

Data collection was carried out via interviews with variable durations (between 20 and 70 minutes). The recruitment and interview period occurred between June 2024 and February 2025. Scripts were used with open questions about long-term hospital stays using the following thematic axes: (1) perceptions of long-stay patients, (2) challenges and impacts on care and follow-up, (3) relationship with the health team, (4) recommendations and strategies in care delivery and (5) impacts on personal and professional life. The questions were developed by the research team and tested in a pilot interview, and subsequently adapted to ensure the clarity and relevance of the instrument.

The number of interviews was determined according to the principle of theoretical saturation. Data collection and preliminary analysis were conducted concurrently. After each set of interviews, the research team reviewed the accounts and compared them with the codes and analytical categories already identified. Saturation was considered to have been reached when a consistent pattern emerged, the content became repetitive, and no new codes, analytical categories, or relevant aspects related to the study objective were identified. Recruitment was then discontinued. The final sample of 18 participants was considered sufficient because it included family caregivers and health professionals from different professional categories and provided sufficiently detailed accounts to support the analytical themes. Eight family caregivers of patients with long-term hospital stays were interviewed, with two interviews conducted in the hospital setting and six in the participants’ homes. Ten health professionals were interviewed, including one physician, two nurses, two psychologists, two social workers, and two health managers. All interviews were recorded and transcribed verbatim.

### Reflexibility of the researchers

The interviews were conducted by two master’s students (T.A.G., R.N.D), a social work professional (A.B.C), a field researcher (B.L.P), and a PhD-level qualitative researcher with experience in social medicine research (V.A.M), None of the interviewers had any prior relationship with the participants. The study design was carried out by T.A.G., V.A.M., J.A.S., E.S.C., F.G.M., and W.M. Data analysis and curation were carried out by T.A.G., J.S.C., M.G.F., G.L.A., F.N.O., V.A.M., and W.M. The PhD-level researchers V.A.M, J.A.S, F.G.M, and W.M. have prior experience in qualitative research involving health professionals and vulnerable populations in the Brazilian Amazon.

The research team included members with clinical, social, and qualitative research backgrounds. These different disciplinary perspectives influenced the formulation of interview questions and the interpretation of participants’ narratives. To address this, coding decisions and theme development were discussed collectively during research meetings. Divergent interpretations were reviewed through comparison with the interview transcripts and discussed until consensus was reached.

### Data analysis

Clinical and sociodemographic data from medical records were summarized using absolute and relative frequencies to characterize the study context. The interview transcripts constituted the qualitative data corpus and were subjected to thematic analysis. Thematic analysis was conducted according to Braun and Clark [18] approach using a deductive coding strategy. Andrew and Powell [15] concept of social admission was used as the main conceptual framework, guiding the coding of issues related to the absence of caregivers, fragile family and social support networks, unsuitable conditions for home care, barriers to hospital discharge, and limitations in health and social care services. These dimensions guided the development of the initial codes and the organization of the themes. Atlas.ti software was used to organize codes and themes (. The analytical process involved: (1) familiarization with the data, (2) initial coding, (3) review and refinement of codes, (4) grouping of codes into themes, and (5) interpretation and construction of the final themes. Different interpretations were discussed collectively and resolved by consensus.

## Results

### Patient characteristics

Table 1 presents the characteristics of the long-term hospitalized patients at the institution, including sociodemographic and clinical aspects such as age, comorbidities, marital status and hospital outcomes. Patients were categorized based on the presence or absence of caregivers during hospitalization.

**Table 1:** Sociodemographic characteristics of long-term hospitalized patients in a referral hospital for infectious diseases in the Brazilian Amazon.

Variable	Without a Companion (N = 27)	With a Companion (N = 24)
**Age (Years)**		
0-19	3	1
20-39	10	10
40-59	12	10
60-79	1	3
≥80	1	0
**Gender**		
Male	22	16
Female	5	8
Indigenous		
No	26	23
Yes	1	1
**Education**		
Incomplete elementary school	16	10
Complete elementary school	2	0
Incomplete high school	2	3
Complete high school	4	9
Incomplete higher education	1	0
Complete higher education	0	1
Unknown	2	1
**Marital State**		
Single	24	13
Common-law marriage	0	1
Married	3	7
Divorced	0	3
**Origin**		
Manaus	20	19
Other municipalities of the Amazonas state	6	4
Other states	1	1
**Outcome**		
Transfer	16	4
Death	10	5
Discharge	1	15

*Tefé (1), Urucará (1), Tabatinga (1), Presidente Figueiredo (1), Manacapuru (2), Iranduba (1), Borba (1), Beruri (1), and Boa Vista do Ramos (1).

**Other states (2).

Regarding the profile of caregivers, it was observed that in 11 cases (45.8%), care was shared among two or more family members, indicating an attempt to alternate responsibilities throughout the entire hospitalization period. In the remaining 13 cases (54.2%), care was provided exclusively by one person, predominantly the mother (46.1%), followed by the daughter (15.4%), sister (15.4%), sister-in-law (7.7%), partner (7.7%) or brother (7.7%). Hospital accompaniment was primarily carried out by women (84.6%).

Table 2 presents the distribution of reasons for hospitalization for patients with and without a diagnosis of HIV. Most cases involve HIV-positive patients, accounting for 76.5% of hospitalizations. All the HIV-positive patients were in the AIDS stage of the disease. The most frequent causes of hospitalization among these patients included neurotoxoplasmosis (17.7%), extrapulmonary tuberculosis (11.7%), and pulmonary tuberculosis (9.5%). In contrast, HIV-negative patients represented 23.5% of hospitalizations, with hepatitis C being the leading cause of hospitalization (9.5%).

**Table 2:** Distribution of reasons for hospitalization in long-term hospitalized patients, comparing HIV-positive and HIV-negative patients, with the relative frequency of each admission diagnosis.

HIV status	Reason for hospital admission	Frequency (%)
**HIV-positive patients (n = 39)**	Neurotoxoplasmosis	9 (17.7%)
	Extrapulmonary tuberculosis	6 (11.7%)
	Pulmonary tuberculosis	5 (9.5%)
	Cytomegalovirus retinitis	3 (5.8%)
	Cytomegalovirus encephalitis	2 (3.8%)
	Hepatitis C	1 (2.0%)
	Chronic diarrhea	1(2.0%)
	Cocaine-related psychiatric disorder	1(2.0%)
	Community-acquired bacterial pneumonia	1(2.0%)
	Cytomegalovirus esophagitis	1(2.0%)
	Epstein-Barr virus meningoencephalitis	1(2.0%)
	Herpes simplex	1(2.0%)
	Infectious laryngitis	1(2.0%)
	Leptospirosis	1(2.0%)
	Liver cirrhosis	1(2.0%)
	Malignant neoplasm of the anus	1(2.0%)
	Neurosyphilis	1(2.0%)
	Recurrent urinary tract infection	1(2.0%)
	Rubella	1(2.0%)
	**Subtotal**	**39 (76.5%)**
**HIV-negative patients (n = 12)**	Hepatitis C	5 (9.5%)
	Hepatitis B and D	1(2.0%)
	Lepromatous leprosy	1(2.0%)
	Recurrent erysipelas	1(2.0%)
	Pemphigus foliaceus	1(2.0%)
	Lymphatic filariasis	1(2.0%)
	Bacterial meningitis	1(2.0%)
	Snakebite envenomation	1(2.0%)
	**Subtotal**	**12 (23.5%)** **51 (100.0%)**

During the long-term hospital stay, many patients developed new clinical conditions or complications that impacted their progress. Table 3 presents the distribution of diseases acquired or diagnosed during hospitalization, differentiating the cases according to HIV serological status.

**Table 3:** Distribution of diseases acquired or diagnosed during hospitalization among patients with long-term hospital stay, categorized according to HIV serological status.

HIV status	Diagnoses during hospitalization*	Frequency (%)*
**Positive (n = 39)**	Pressure injury	31 (60.8%)
	Hospital pneumonia	10 (19.6%)
	Hypertension	8 (15.7%)
	Diabetes mellitus	7 (13.7%)
	Neurotoxoplasmosis	6 (11.8%)
	Pulmonary tuberculosis	6 (11.8%)
	Hospital urinary tract infection	5 (9.8%)
	Covid-19	4 (7.8%)
	Citomegalovirus retinitis	4 (7.8%)
	Sepsis with pulmonary focus	3 (5.9%)
	Herpes zoster	3 (5.9%)
	Pneumocystosis	3 (5.9%)
	Severe anemia	2 (3.9%)
	Sepsis with urinary focus	2 (3.9%)
	Genital herpes	2 (3.9%)
	Pleomorphic parotid adenoma	1 (2.0%)
	Severe malnutrition	1 (2.0%)
	Chronic diarrhea	1 (2.0%)
	Chronic obstructive pulmonary disease	1 (2.0%)
	Hepatic encephalopathy	1 (2.0%)
	Pulmonary emphysema	1 (2.0%)
	Chronic gastritis	1 (2.0%)
	Digestive bleeding	1 (2.0%)
	Hepatitis C	1 (2.0%)
	Inguinal hernia	1 (2.0%)
	Histoplasmosis	1 (2.0%)
	Acute renal failure	1 (2.0%)
	Malaria	1 (2.0%)
	Malignant neoplasm of the liver	1 (2.0%)
	Neurotuberculosis	1 (2.0%)
	Neurosyphilis	1 (2.0%)
	Osteomyelitis	1 (2.0%)
	Pityriasis versicolor	1 (2.0%)
	Unspecified pneumothorax	1 (2.0%)
	Idiopathic thrombocytopenic purpura	1 (2.0%)
	Sepsis of undetermined focus	1 (2.0%)
	Syphilis	1 (2.0%)
	Dementia	1 (2.0%)
	Extrapulmonary tuberculosis	1 (2.0%)
	Progressive multifocal leukoencephalopathy	1 (2.0%)
**Negative (12)**		
	Pressure injury	8 (15.6%)
	Hypertension	3 (5.8%)
	Blastomycosis	1 (2.0%)
	Covid-19	1 (2.0%)
	Squamous cell carcinoma	1 (2.0%)
	Liver cirrhosis	1 (2.0%)
	Pleural effusion	1 (2.0%)
	Calcium metabolism disorder	1 (2.0%)
	Ascending aortic ectasia	1 (2.0%)
	Recurrent erysipelas	1 (2.0%)
	Leprosy erythema nodosum	1 (2.0%)
	Malignant neoplasm of the liver	1 (2.0%)
	Surgical site infection	1 (2.0%)
	Liver failure	1 (2.0%)
	Hospital pneumonia	1 (2.0%)
	Chronic cutaneous porphyria	1 (2.0%)
	Carotid pseudoaneurysm	1 (2.0%)
	Sepsis with urinary focus	1 (2.0%)
	Sepsis with pulmonary focus	1 (2.0%)
	Dementia	1 (2.0%)
	Drug-induced Cushing’s syndrome	1 (2.0%)
	Pulmonary tuberculosis	1 (2.0%)

*A single patient may present with more than one diagnosis during hospitalization.

### Emerging themes

From the thematic analysis of the interviews conducted with the caregivers and the health professionals involved in the care of the patients during the long-term hospital stay, it was possible to identify five main themes: (1) social hospitalization and institutional dependence, (2) dynamics of family and social ties, (3) the routine of care and the impacts on the lives of caregivers (4) clinical complexity and obstacles that impede hospital discharge and (5) the need for public policies and infrastructure. The themes are presented below:

#### Social hospitalization and institutional dependence.

During the long-term hospital stay, the hospital became their home, especially in cases of lack of family support and social vulnerability of the patient. Thus, we observed the naturalization of this condition both by patients and their families and by health professionals. The caregivers, for example, adopted the hospital’s routine, internalizing the schedules and practices related to care and institutional functioning:

*- “I got into the rhythm and, every day, I followed the routine of the hospital and not that of outside... I woke up at five in the morning, at the same time, and did the same things... the routine has always been the same.” (Son, caregiver for 5 years)*

*- “...because the caregiver ends up following the routine of care, of all the issues related to the hospital routine (Health professional 2)*

The prolongation of the patients’ hospitalization also extends the caregivers’ stay in the hospital, compromising their emotional bonds and social commitments outside the institution. Because they remain in the hospital environment for such long periods, many are informally referred to as “residents,” in reference to the fact that they are practically living in the hospital. Furthermore, the workload imposed on these caregivers is frequently highlighted by health professionals, revealing the fragility, or even absence, of broader support networks.:

- *“Because practically my father and I became residents here at the Tropical Hospital. They even called me a resident of the Tropical [in relation to spending a lot of time at the institution].” (Son, caregiver for 5 years)*

*- “And his father took care of him. His father came every day. Every single day. His father put his life on hold. He spent the day and night living here...)” (Health professional 9)*

*- “In fact, most caregivers do not take turns. That’s very interesting. You have to quantify that there. But, in a very experiential way, most do not take turns. The one who volunteers to stay here is usually the one who will stay for the whole time” (health professional 3)*

In this context, the caregivers assumed care that is typical of health professionals, as a strategy for creating and maintaining links with professionals and the hospital

*- “Anything that I saw that was abnormal. Sometimes, when it was time to move and something came loose, I had the materials there. I didn’t even need to call someone. I fixed it myself, got everything right” (father, caregiver for 4 years)*

*- “Usually the girls [health professionals] always ask me for help, but since I have already created some techniques, I have already learned everything, I really tried to learn. I integrated myself to learn, because I said that if one day my brother goes home, I already know how to do something for him, because obviously if he went home, the person who would take care of him would be me” (Sister, caregiver for 2 years)*

*- “And we created a friendship between us [myself and the health professionals] because there was a reciprocity, both on their part [health professionals], and on my part (...). Got it? We chatted, and I helped with what I could, you know?” (Sister, caregiver for 2 years)*

For patients in situations of social vulnerability, long-term hospital stays were a way of guaranteeing food, hygiene, medication at regular times and even housing, even though hospital care was already unnecessary. Some caregivers kept their family members hospitalized because they needed to work, and the patient did not have a wider support network:

*- “Often, they [the family members], themselves, prefer that they stay here because it is a matter of food. Here, you have food at the right time, you have medication at the right time. There is a bathroom." (Health professional 7)*

*- “... a dependent person at home, who does not walk without help and such, and the person who has little money has to work and such, all this makes it difficult for him to return to the family. And then the patient stays in the hospital, occupying the bed.” (Health professional 9)*

The same happened with socially vulnerable and unaccompanied patients. In this study, we mean homeless people with neurological deficits, from the interior of the state and patients with AIDS:

- *“Today she is in bed and is already in social care. [...] The patient is discharged, but is in a social institution, because we also have no way to remove this patient from here and put him on the street, because they are totally dependent and bedridden patients who use a wheelchair.” (Health professional 1)*

*- “He has to have a caregiver, but how is he going to have a caregiver if he’s homeless?” (Health professional 2)*

#### Dynamics of family and social ties.

During prolonged hospitalizations, we observe the weakening of family ties and the formation of new ties in the hospital environment. The fragility of these bonds is also an obstacle that impedes discharge, especially in cases requiring intensive care. In some cases, family members and professionals use their own resources to meet health system needs and support patients in situations of abandonment (unaccompanied):

- *“For most of the patients that were bedridden, I went out and bought diapers, we bought hygiene supplies... I said, ‘Guys, this isn’t just for my brother, it’s for all the patients there.” (Sister, caregiver for 2 years)*

- *“Sometimes the nursing technicians had to take money out of their own pockets and give it to the patient... they had a collection, they helped...” (Son, caregiver for 5 years)*

Despite these support networks, the absence of permanent family support – for caregivers and patients – is still predominant:

*- “My mother is already dead. My sister is also dead. It’s just the two of us now. There’s no one else.” (Brother, caregiver for 1 year)*

*- “There are patients who die, and the family does not even know, they don’t come to accompany the patient, nor know how he is, and he dies and stays there in the mortuary.” (Health professional 7)*

Throughout the hospitalization, patients, caregivers and professionals established bonds of affection and trust:

*- “The routine is that they are residents, and we get more attached to them, right? [...] With the time they stay, you will get more intimate and begin to suffer a lot with the patient.” (Health professional 6)*

*- “The person stays in the hospital, isolated, with the patient... if you don’t have good friendships or you don’t have entertainment, you can freak out.” (Father, caregiver for 5 years)*

However, the fragility of family and social ties imposes itself as a concrete obstacle to returning home. The stories reveal bonds broken by abandonment, violence or the family’s inability to provide the necessary care:

*- “The family refuses, claims that they have no bond, that they have no way to take them in.” (Health professional 1)*

*- “That person who has no bond is already a more difficult case... it has happened in the domestic abuse issue, domestic violence issue.” (Health professional 1)*

*- “His father’s parents died, his grandmother and grandfather, his uncles threw him out of the house... then he came to ask for shelter, brought himself back [to the hospital].” (Grandmother, caregiver for 1 year)*

Social vulnerability is also related to the weakening of family ties and even complete abandonment. Extreme poverty and lack of family support are frequently mentioned aspects:

*- “They have no conditions for offering care. They can’t afford to buy a diaper. They can’t stop working to take care of someone.” (Health professional 10)*

*- “There is no point in asking someone to be okay... If everything else is lacking. The basics are lacking. If you don’t have a home to return to...” (Health professional 4)*

*- “He’s been with us for six or seven months now. He’s a patient you can take home. But the family does not accept him. They say they can’t care for him.” (Health professional 6)*

#### The care routine and the impacts on the lives of caregivers.

The long-term hospital stay not only affected the patients, but also had repercussions on the lives of the caregivers in emotional, physical and social aspects. The reports below highlight how the care routine transformed the daily lives of the caregivers. Some have made caring for their inpatient family the biggest part of their lives:

*- “Because I stopped living. That I live for him, by taking care of him.” (Sister, caregiver for 2 years)*

*- “Imagine the psychological impact. You leave all your life behind, that sacrificial love for that person. [... You don’t sleep in a bed; you always sleep in an armchair.” (Health professional 4)*

*- “We realize that this family member who makes the decision to accompany a patient who will remain without a discharge date... they end up giving up a lot of things.” (Health professional 2)*

*- “I often felt like giving up... ‘I’ll stop it. Why am I here, guys? I have my life out there.” (Sister, caregiver for 2 years)*

Faced with the progressive deterioration of the health of patients with no prospect of recovery, some caregivers even reported the desire to shorten the suffering through the death of the family member:

*- “Let him rest in peace. Let him stay there without having to do anything, I don’t want him intubated anymore. That’s enough. That’s good enough.” (Sister, caregiver for 2 years)*

*- “He’s been in the hospital for a long time and he knows that patient is not coming back. I was waiting for God’s will (...) (Mother, caregiver for 1 year)*

#### Clinical complexity and obstacles that impede hospital discharge.

In addition to social vulnerability and lack of family support, the long-term hospital stay also resulted from the clinical complexity of the hospitalized patients. In this study, we found cases of multiple comorbidities, irreversible sequelae, permanent invasive devices and high dependence on specialized medical care. The reports point to severe and chronic clinical conditions, such as tracheostomy, use of enteral catheter, extensive pressure injuries, recurrent bacterial infections and neurological impairment:

- *“It was a patient that had atrophied lower limbs, had pressure injuries. It was a patient who was tracheostomized, probed (...). He was a real resident, and this lasted for five years.” (Health professional 6)*

- *“There were some bacterial infections there. Multidrug-resistant bacteria. The intravenous medication even had to stay. So, I think she’s been there more than a year.” (Health professional 9)*

- *“Those who stay a long time, who are patients with sequelae, who use trachea, they [family members] are afraid to take them home.” (Health professional 1)*

Even when there are family members willing to take them home, the complexity of the necessary care, as well as the respective costs, is a significant barrier to the success of hospital treatments in a home environment:

*- “But, if I really had support, I would take him home, but we don’t have support. (...) The [patient] needs an ICU. Honestly, if I had the conditions, my brother would be back at home by now. (...) So, he has to have all the support. His bed has to be special, right? And that is difficult. You set up an ICU like that, save money to set up an ICU like that.” (Sister, caregiver for 2 years)*

*- “If I took him home, he still wouldn’t have his support (...), I don’t know if he would like it after three years in there, you know? Adapting at home (...), then I was going to do what? Just take care of him? Because it’s a lot [demand for care], right? And I don’t know how I would move him, knowing that his food is complicated, because it is special, I don’t know well, diapers, these things. (Son, caregiver 5 years).*

Another impediment to hospital discharge was the lack of places in long-term care units or shelters and the infrastructure needed for patient transfer:

- *“And what else do you see around? Full shelters, crowded, there is nowhere to put more people.” (Health professional 2)*

- *“And I’m waiting for a vacancy to go there. But there at the Foundation it is like this: Only one gets in when one dies.” (Brother, caregiver for 1 year)*

- *“It is not possible to transport a person who needs a specific diet by boat. Even in a speed boat. The time the speed boat takes is much longer than it would be by plane.” (Health professional 8)*

Another major obstacle to discharge is the stigma associated with HIV/AIDS. Many patients face rejection by their families after diagnosis, which directly contributes to a prolonged hospital stay:

*- “Newly diagnosed patient, so she was kicked out of her son’s house because of HIV.” (Health professional 1)*

*- “When everyone found out that he was in this situation, everyone turned away from him” (Mother, caregiver for 5 years)*

*- “My wife doesn’t want him to live at home...] when you have a virus, everyone thinks that just talking, holding hands, [...] the others will catch it.” (Brother, caregiver for 1 year).*

Stigma contributes not only to family estrangement, but also hinders social reintegration, even when the patient is clinically stable. Often fear, misinformation and prejudice contribute to exclusion:

*- “Because there is still the issue of prejudice, of knowledge, even, from some professionals. Because I have already taken a patient to the shelter and when she saw that he was HIV positive, the prejudice [refusal to receive him] was notorious” (Health professional 1)*

*- ‘So, I had that estrangement by my family. Then came the disease [HIV/AIDS] and now the complication came. […] “So now I really don’t want to be at home” [reproduction of family statements] So we still have a lot of this linked to our patient” (Health professional 11)*

#### The need for public policies and infrastructure.

The long-term hospital stay is affected by the fragilities of the infrastructure and the scarcity of adequate public policies for social hospitalization and the respective discharge from hospital. Some caregivers, for example, need government aid to meet basic needs related to the health care of their family members:

*- “It helps a lot (refers to government financial aid). Because if it wasn’t for that help, I wouldn’t be able to [...] stay with him [providing the care in the hospital]. [...] I buy cleaning supplies, the ointments you need to buy, there are some ointments that are very expensive.” (Sister, caregiver for 2 years)*

*- “Doing the math, it was forty thousand that I paid. Forty thousand. For those who earn a minimum wage. (...), But I thank God for what he earned, that there are many people there who do not even earn money” (Mother, caregiver for 1 year)*

In addition, essential supplies are occasionally lacking, such as medicines, personal protective equipment and basic items for daily care, which end up being paid for by the caregivers themselves:

*- “There is no medication. One day I had no medication. Lack of medication. (Mother, caregiver 4 years)*

*- “Sometimes I have to pay for it out of my own pocket, I buy it myself if I want to see an improvement. [...] I would buy a box of gloves, masks, all of that stuff. [...] I also bought a lot of diapers, because he got fat, so they didn’t fit, but I used them [amended] the medium (...) and the large, extra-large (...).” (Mother, caregiver for 1 year)*

*- “There was a time that I bought them, because there wasn’t any at the hospital. Sometimes too... I would buy diapers. It wasn’t for long, no. But sometimes there were days that I didn’t have any. And then I bought them, because I always saved his money (Father, caregiver for 6 years)*

The prolonged stay of these patients also affects the management of hospital beds:

*- “Today, if I have five patients here inside the hospital, there are five beds that I cannot use.” (Health professional 11)*

*- “This greatly complicates the turnover and hospitalization of other patients.” (Health professional 9)*

*- “Sometimes it impacts things, leaving the emergency room overcrowded.” (Health professional 11)*

Given this scenario, the health professionals expressed the need to create specific public policies for the reception and monitoring of patients in social hospitalization after hospital discharge. The fragility of intermediate infrastructure and the scarcity of public policies, such as the creation of shelters and institutions focused on long-term care, is pointed out as a gap in the health system:

*- “I believe that if there was at least one specific shelter for these people [...], I think it would be more interesting than being in the hospital.” (Health professional 1)*

*- “Because that way, he doesn’t need a hospital per se, he needs a shelter, a place to live.” (Health professional 11)*

*- “There could be, after hospital discharge, a team that followed-up this patient for at least 3 months [referring to a home follow-up].” (Health professional 2)*

**Fig 1:**
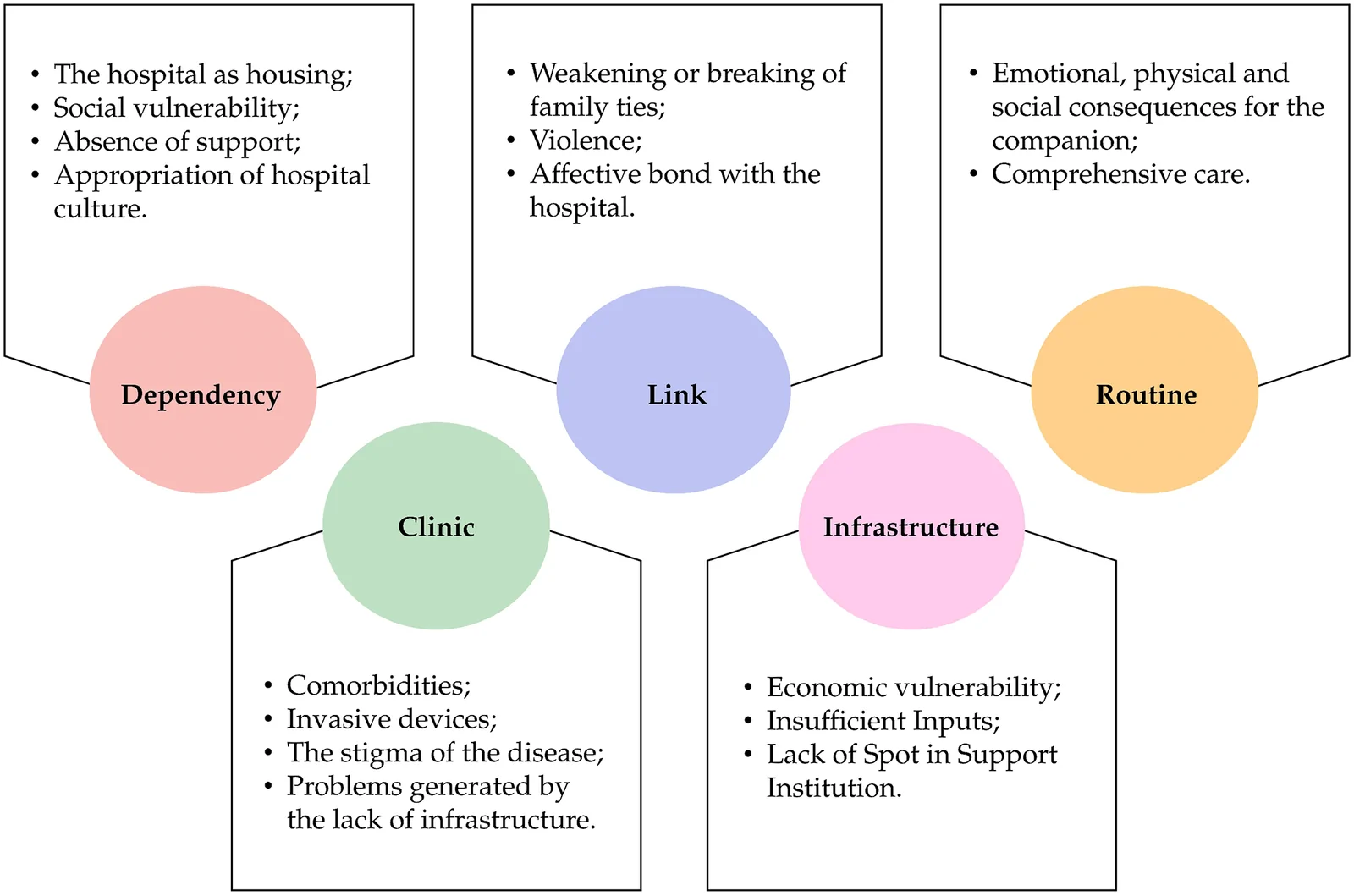
Key subtopics identified within the five main themes of the thematic analysis on prolonged hospital stays.

The emerging themes from the analysis reflect systemic gaps in the care of patients with prolonged hospital stays in our study context. Their core elements are visually organized in Fig 1, providing a comprehensive overview of the multiple barriers to hospital discharge and social reintegration.

## Discussion

In this study, most of the long-term hospital stay patients were people with low socioeconomic conditions, low education levels and with weakened or even broken family ties. Most of these patients needed social hospitalization. The study by Ghost et al. [19] showed that patients with lower socioeconomic status and low education levels tend to remain hospitalized longer due to difficulties in accessing the resources necessary for home care, the absence of broader social and family support networks, and also because low education levels often equals low levels of health literacy.

The low proportion of Indigenous patients may also be related to the specific support network available to this population. In Amazonas, Indigenous Health Houses (CASAI) provide accommodation, assistance, and support to Indigenous patients referred for specialized care and, when necessary, to their caregivers. This structure may facilitate continuity of care and reduce prolonged hospitalization related exclusively to the lack of family or social support [20].

The diagnoses observed mostly involved infectious diseases associated with HIV/AIDS, such as neurotoxoplasmosis, cytomegalovirus infection, tuberculosis, and pneumocystosis. We also identified tropical diseases, including leprosy, malaria, leptospirosis, and histoplasmosis, as well as complications associated with prolonged hospitalization, particularly pressure injuries and healthcare-associated infections. Al-Shammari et al. [21] and Huang [22], when analyzing the impact of chronic morbidities and infections with neurological involvement on functionality and length of stay, showed that the progressive accumulation of diagnoses throughout hospitalization may significantly compromise patients’ physical and cognitive abilities. This functional deterioration may worsen the clinical condition, hinder rehabilitation and discharge, and reinforce a cycle of hospital dependence. Complications developed during hospitalization, particularly pressure injuries, hospital-acquired pneumonia, and urinary tract infections, may further increase immobility, dependence on care, and the need for additional treatment. Previous studies have demonstrated that hospital-acquired complications and healthcare-associated infections are associated with increased length of stay [23,24].

Long-term hospitalizations that evolved into social hospitalization, due to socioeconomic vulnerability, abandonment by the family and the need for exclusive care in the hospital environment, were recognized by accompanying family members and professionals as a residence status. For Oliver [25] and Mah et al. [26], the term social hospitalization is equivalent to a classification that can reinforce stigmas and naturalize the stay in hospital, making it difficult to implement a dignified and structured therapeutic plan. However, we understand that this phenomenon is related to a broader social process that involves socioeconomic inequalities, socio-family relationships, the progressive worsening of the clinical picture, and the stigma associated with HIV/AIDS, in the case of this study, in addition to structural problems of the health system itself and the scarcity of public policies that guarantee access to fundamental rights for the dignity of life.

In this sense, Andrew and Powell [15] demonstrated that social hospitalizations are markers of multifactorial vulnerability, related not only to medical conditions, but also to the structures of the health systems that manage these patients. For Edwards et al. [5], without the implementation of healthcare protocols that assist these patients in issues related to logistics with viable solutions, the hospital becomes an alternative to to maintain continuity of healthcare.

The fragility of family ties and abandonment constitute an obstacle to a safe discharge, making it unfeasible to organize care outside the hospital and reinforcing the practice of social hospitalization. Our findings were similar to those in the study by Louis Simonet et al. [27] and Weaver & Weaver [28], because the less support there is available, the longer the hospitalization time, especially in the absence of intermediate institutions, such as long-term care facilities, and policies aimed at home care. Scientific studies on the subject reinforce that social and family reintegration after hospital discharge faces challenges such as dependence on advanced life support, risk of complications, and the family’s refusal to take responsibility for highly dependent patients [12,13,29].

In addition, patients from municipalities in the interior of the state may face greater difficulties in maintaining family support during prolonged hospitalizations because of long distances, travel costs, and limitations on caregivers’ ability to remain in Manaus. These factors may contribute to the weakening of family and community ties and make it more difficult to organize safe care after discharge [27–29].

In 54.2% of hospitalizations, the patient’s hospital follow-up was performed by a single family member. 84.6% were women, respectively, mothers, daughters, sisters and sisters-in-law. Moser and Dal Prá [30] point out that the act of caring almost always falls on a single woman in the family, usually motivated by feelings of moral obligation, guilt or by naturalized social norms. Although the present study did not consider markers of race and class, it is worth noting that Gonçalves and Paiva [31] indicated that care and follow-up in hospital admissions in the Brazilian Amazon is mostly performed by poor black women.

The routine of care in the hospital can impact the life of the caregivers, causing physical and emotional exhaustion and motivating the renunciation of personal projects. In some cases, the impact is significant to the point of provoking in the caregiver the desire that this situation be abbreviated with the death of the hospitalized family member, understood as redemption. Some studies indicate that health professionals live an ethical dilemma in the face of the lack of alternatives to manage patients with indeterminately prolonged care and the physical and psychological illness of caregivers through their extensive experience in the hospital environment [32–35].

The stigma associated with HIV/AIDS and the presence of multiple comorbidities directly caused abandonment by the family and contributed to the social hospitalization of the patients included in this study. Schilkowski et al. [36] describe that living alone or in shelters and reception institutions is a common reality among people who develop a clinical picture of AIDS. For Micallef et al. [37], patients with socially stigmatized diseases tend to have their needs neglected, making it difficult to prepare discharge plans and the possibility of transfer to intermediate institutions or to return patients to the family nucleus.

The naturalization of abandonment reflects the appropriation of the hospital space as a long-term care setting for patients rejected by their families. Foucault [38] describes the historical trajectory of the hospital as a place of segregation of the poor, the mentally ill, beggars, and people with leprosy, highlighting that, over the centuries, the hospital also fulfilled a social function of sheltering socially excluded individuals. In this sense, under the discourse of social assistance, the hospital may be perceived as a place of shelter and residence, reinforcing practices of exclusion.

In the hegemonic discourse, the prolonged stay of these patients has a direct impact on hospital management, as reported by some of the professionals regarding the turnover of beds, the flow of new admissions and the overcrowding of emergency rooms; findings similar to others described in the scientific production regarding the area [10,39]. However, Ornellas [40] warns that understanding the hospital under the logic of capitalist production implies treating care as a commodity, requiring users to adapt to performance and efficiency criteria. In addition to conceiving the patient as an obstacle to productivity, this ideological conception ignores the constituent reasons for the phenomenon of long-term hospital stays.

Our findings reinforce the need for public policies aimed at continuity of care outside the hospital environment. The absence of vacancies in intermediate institutions of reception, home support and effective government initiatives keeps patients hospitalized for longer than what is necessary. Rojas-García et al. [41] describe that the lack of articulation between the health and social care sectors constitutes one of the main obstacles that impede discharge from hospital, preventing the construction of safe and sustainable alternatives outside the hospital environment. These issues generate impacts for both patients and services, being a structural gap recognized by the Brazilian Ministry of Health [42].

Taken together, these results indicate that long-term hospitalization was not sustained by isolated clinical or family-related factors. Rather, it resulted from the interaction between social vulnerability, fragile support networks, functional dependence, stigma, and gaps in health and social care systems. From the perspective of social admission (15), these elements collectively limited the possibility of safe discharge and reinforced institutional dependence.

## Limitations

This study focused on the experiences, meanings, and perceptions of health professionals and caregivers, which constituted the central object of the qualitative inquiry and were interpreted within the social and institutional context in which they were produced. The direct perspectives of patients could not be included because many had already died or had clinical and/or cognitive impairments that prevented their participation. This limited a broader understanding of their experiences during prolonged hospitalization.

Anxiety, depression, and post-traumatic stress disorder were not systematically assessed because no specific psychological or psychiatric screening instruments were used. Furthermore, the clinical data were retrospectively obtained from medical records, in which these conditions may have been underdiagnosed or incompletely documented. Therefore, their absence from the results should not be interpreted as evidence that these conditions were not present among patients with long-term hospital stays.

This study was conducted in a single referral hospital in the Amazon region, whose geographical, social, and structural characteristics are particular to this context. Therefore, the findings should not be directly generalized to other institutions or regions of the country. Nevertheless, the results contribute to the understanding of a phenomenon that remains underexplored and broaden the debate on the need for greater visibility of prolonged hospital stays, improved support for hospital discharge processes, and the reduction of inequalities in access to health care in contexts of high vulnerability.

## Conclusion

This study, a pioneer in the context of the Brazilian Amazon, investigated why some patients remain hospitalized for long periods in an Amazonian hospital, even after achieving the clinical state needed for discharge. It has been identified that many of them live with HIV/AIDS and face not only the complexity of the disease, but also the social stigma it carries. This prejudice often results in abandonment by the family and lack of support for care at home. In addition, many families reported not having the financial, structural or emotional conditions to ensure the basic care that these patients need. In the absence of effective public policies and an adequate support network, the hospital ends up becoming the only possible place for these people to live. Our results demonstrate the need for public policies that integrate health, social assistance and protection of this vulnerable population, guaranteeing the right to continuity of care outside the hospital environment.

Possibly, the response to long-term hospital stays requires integrated strategies between the different health sectors. This includes strengthening intersectoral networks, creating intermediary care institutions, addressing the stigma of diseases such as HIV/AIDS, and providing effective support to caregivers. However, such measures, although essential, do not account for the complexity of the problem. The long-term stay is related to persistent social inequalities that limit access to health, education, housing and social protection. Without addressing these conditions, any solution tends to be palliative and unable to break the cycle of vulnerabilities that keeps certain groups on the sidelines of the most basic rights.

## Supporting information

S1 Text COREQ. Consolidated Criteria or reporting qualitative research.
(DOCX)

S2 Text Codebook. Codebook used in the qualitative analysis of interviews for the study.
(PDF)
