# Supplementary material for: The hospital as the only place to live: Long-term hospital stays in a tertiary hospital for infectious diseases in Manaus, Brazilian Amazon
Source: PLoS One. 2026 Sep 8;21(9):e0357832. doi: 10.1371/journal.pone.0357832 (PMC13552958; doi:10.1371/journal.pone.0357832)
Supplement: Supplementary file 1 [file pone.0357832.s002.pdf]

Código	Frequência
1 -Adaptação a rotina Hospitalar	3
2 -Assistência Voluntária	11
3 -Ausência de Suporte Familiar	8
4 - Carga de trabalho do acompanhante	24
5 - Complexidade clinica	23
6 -Criação de vinculos	12
7 - Dependência de Auxílios Governamentais	10
8 -Dependência de Cuidados Exclusivamente Hospitalares.	7
9 Barreiras para Transferência Domiciliar e Hospitalar	26
10 -Esperança, apesar do prognóstico negativo	1
11 -Estigma	28
12 -Ausência de Rede de Suporte Pós-Alta	22
13 - Falta de Insumos	15
14 - Cuidados Hospitalares	12
15 Fragilidade dos Vínculos Familiares e Sociais	34
16 - Impacto físico e mental	18
17 - Gestão de Leitos de Longa Permanência	3
18- Moradia Hospitalar	28
19 - Descontinuidade no tratamento do HIV.	9
20 - Desejo de Abreviação do Sofrimento mediante a Morte.	8
21 -Recomendações de Melhoria	13
22 - Vulnerabilidade Social	29
23 Convivência e Aprendizado (Adaptação da Rotina Hospitalar)	7
24 Humanização do cuidado (Atenção e cuidado institucional)	15
25 Quadro Clinico Instável (Complexidade Clinica)	24

Comentários
Adaptação as normas e rotinas hospitalares.
Ato de prestar ajuda ou serviços de forma espontânea e sem remuneração, visando o bem-estar do pacient
Falta de suporte familiar associados ao acompanhamento durante a internação.
Atividades realizadas pelo acompanhante durante a internação.
Presença de múltiplas condições de saúde, exigindo cuidados especializados, manejo interdisciplinar e supo
Processo de estabelecer conexões emocionais, sociais ou profissionais , baseado na confiança, empatia e int
Dependência de auxílios governamentais para manutenção dos cuidados durante a internação.
Dependência da estrutura hospitalar para medicação, alimentação, assistência contínua ou suporte social, s
Barreiras na transição do paciente entre unidades de saúde, devido a fatores clínicos, burocráticos ou falta
Expectativas acerca da melhora e alta hospitalar.
Rótulo social negativo que leva ao preconceito, discriminação e exclusão de indivíduos devido a uma condiç
Falta de recursos e estrutura familiar e/ou profissionais da assistência social e saúde que possibilite a contir
Escassez de materiais, equipamentos ou medicamentos essenciais para a assistência adequada
Cuidados em todos os níveis de atenção hospitalar, incluindo aqueles próprios da assistência social.
Rupturas ou fragilidades dos vínculos familiares e sociais, afetando a socialização, a complementação de rec
Impactos na saúde física e mental dos acompanhantes durante toda a internação.
Estratégias de gestão e impactos causados pela ocupação prolongada dos leitos.
Ampliação involuntária das responsabilidades institucionais, incluindo, dentre os cuidados hospitalares,   a r
Descontinuidade do paciente nos cuidados relacionados ao tratamento do do HIV, bem como no acompanh
Diante de um prognóstico irreversível, o acompanhante verbaliza o desejo de que o sofrimento do paciente
Recomendações acerca de como manejar esses pacientes de longa permanência.
Condição de fragilidade socioeconômica que limita o acesso a direitos básicos, aumentando a exposição a ri
Relacionado a vivencias a aprendizados durante a internação.
Diz respeito a como era realizado a humanização do cuidado para com os pacientes e acompanhantes.
Refere-se ao estado clinico do paciente durante a longa permanência hospitalar

de suporte (pegar falas relativas a alta e coloca no código Dependência Hospitalar)
