# Supplementary material for: The hospital as the only place to live: Long-term hospital stays in a tertiary hospital for infectious diseases in Manaus, Brazilian Amazon
Source: PLoS One. 2026 Sep 8;21(9):e0357832. doi: 10.1371/journal.pone.0357832 (PMC13552958; doi:10.1371/journal.pone.0357832)
Supplement: Supplementary file 2 [file pone.0357832.s001.docx]

**Consolidated criteria for reporting qualitative studies (COREQ): 32-item checklist - The Hospital as the Only Place to Live: Long-term Hospital Stays in a Tertiary Hospital for Infectious Diseases in Manaus, Brazilian Amazon**

*Developed from:*

*Tong A, Sainsbury P, Craig J. Consolidated criteria for reporting qualitative research (COREQ): a 32-item checklist for interviews and focus groups. International Journal for Quality in Health Care. 2007. Volume 19, Number 6: pp. 349 – 357*

| **No. Item** | **Guide questions/description** | **Reported on Page** |
| --- | --- | --- |
| **Domain 1: Research team and reﬂexivity** |  |  |
| *Personal Characteristics* |  |  |
| 1. Inter viewer/facilitator | *Which author/s conducted the interview or focus group?*  T.A.G / R.N.D / A.B.C / V. A.M | Pg 4 |
| 2. Credentials | *What were the researchers’ credentials?*  Master Student, Doctors. | Pg 4. |
| 3. Occupation | *What was their occupation at the time of the study?*  Master students, PhD students, and qualitative researchers. | Pg 4. |
| 4. Gender | *Was the researcher male or female?*  Some researchers were male, others were female. | Pg 4. |
| 5. Experience and training | *What experience or training did the researcher have?*  The researchers had previous publications experiences with qualitative research in the Brazilian Amazon, as well as training in in-depth interviews. | Pg 4 |
| *Relationship with participants* |  |  |
| 6. Relationship established | *Was a relationship established prior to study commencement?*  None of the participants had an established relationship with an author prior to study commencement. | Pg 4. |
| 7. Participant knowledge of the interviewer | *What did the participants know about the researcher? (e.g., personal goals, reasons for doing the research).*  The first two family members were identified from interactions with the institution's social assistance team.  The indicated professionals were selected from previous experiences with patients. | Pg 4. |
| 8. Interviewer characteristics | *What characteristics were reported about the inter viewer/facilitator? e.g., bias, assumptions, reasons and interests in the research topic.*  No interviewer-related biases were identified. | Pg 4. |
| **Domain 2: study design** |  |  |
| *Theoretical framework* |  |  |
| 9. Methodological orientation and Theory | *What methodological orientation was stated to underpin the study? e.g., grounded theory, discourse analysis, ethnography, phenomenology, content analysis.*  The research team members conducted a deductive thematic analysis, based on an approach to social internments by Andrew and Powell. | Pg 4. |
| *Participant selection* |  |  |
| 10. Sampling | *How were participants selected? e.g., purposive, convenience, consecutive, snowball.*  The approach was intentional, selecting participants based on their availability and relevance of experience with long-term hospitalizations. | Pg 3. |
| 11. Method of approach | *How were participants approached? e.g., face-to-face, telephone, mail, email.*  The first contact with all participants was by phone, following a face-to-face explanation of the research. | Pg 6. |
| 12. Sample size | *How many participants were in the study?*  8 Family Members / Accompanying  9 Health Professionais | Pg 3. |
| 13. Non-participation | *How many people refused to participate or dropped out? Reasons?*  No one has given up on participating in the project. | N/A |
| *Setting* |  |  |
| 14. Setting of data collection | *Where was the data collected? e.g., home, clinic, workplace.*  Some interviews with family members were conducted in the families' residences due to convenience. All interviews with professionals are conducted in the hospital in a closed and silent room. | Pg 3. |
| 15. Presence of non-participants | *Was anyone else present besides the participants and researchers?*  No, only the interviewers were on-site during the interviews. | Pg 3. |
| 16. Description of sample | *What are the important characteristics of the sample? e.g. demographic data, date*  These data are described in the study results | Pg 4. |
| *Data collection* |  |  |
| 17. Interview guide | *Were questions, prompts, guides provided by the authors? Was it pilot tested?*  The questions were developed by the research team and tested in a pilot interview, and subsequently adapted to ensure the clarity and relevance of the instrument. | Pg 4. |
| 18. Repeat interviews | *Were repeat inter views carried out? If yes, how many?*  No | N/A. |
| 19. Audio/visual recording | *Did the research use audio or visual recording to collect the data?*  The interviews were recorded and transcribed without personal identifiers, so that the database could be anonymized | Pg 8. |
| 20. Field notes | *Were ﬁeld notes made during and/or after the inter view or focus group?*  No | N/A. |
| 21. Duration | *What was the duration of the interviews or focus group?*  Data collection was carried out via interviews with variable durations (between 20 and 70 minutes). | Pg 4. |
| 22. Data saturation | *Was data saturation discussed?*  The number of interviews was established according to the principle of theoretical saturation, in which data collection continues until a consistent pattern emerges and additional interviews no longer provide new relevant information | Pg 4. |
| 23. Transcripts returned | *Were transcripts returned to participants for comment and/or correction?*  No. | N/A. |
| **Domain 3: analysis and ﬁndings** |  |  |
| *Data analysis* |  |  |
| 24. Number of data coders | *How many data coders coded the data?*  The initial coding was carried out by three members of the research team and reviewed collectively during team meetings. | Pg 4. |
| 25. Description of the coding tree | *Did authors provide a description of the coding tree?*  Yes. | Supplementary Material |
| 26. Derivation of themes | *Were themes identiﬁed in advance or* *derived from the data?*  The analysis of the interviews and the field notes allowed us to identify five major themes. | Pg 5. |
| 27. Software | *What software, if applicable, was used to manage the data?*  Atlas TI | Pg 4. |
| 28. Participant checking | *Did participants provide feedback on the ﬁndings?*  No. | N/A |
| *Reporting* |  |  |
| 29. Quotations presented | *Were participant quotations presented to illustrate the themes/ﬁndings? Was each quotation identiﬁed? e.g. participant number*  Yes, quotations were presented to illustrate the themes/findings, and each quotation was identified with a participant number. | Pg 5-13. |
| 30. Data and ﬁndings consistent | *Was there consistency between the data presented and the ﬁndings?*  Yes, there was consistency between the data presented and the findings. | Pg 5-13. |
| 31. Clarity of major themes | *Were major themes clearly presented in the ﬁndings?*  Yes, major themes were clearly presented in the Results section using specific sections regarding each theme. | Pg 5-13. |
| 32. Clarity of minor themes | *Is there a description of diverse cases or discussion of minor themes?*  Yes, minor themes were discussed. | Pg 5-13. |
